# A prospective field study of U.S. Army trainees to identify the physiological bases and key factors influencing musculoskeletal injuries: a study protocol

**DOI:** 10.1186/s12891-019-2634-9

**Published:** 2019-06-12

**Authors:** Julie M. Hughes, Stephen A. Foulis, Kathryn M. Taylor, Katelyn I. Guerriere, Leila A. Walker, Amy F. Hand, Kristin L. Popp, Erin Gaffney-Stomberg, Kristin J. Heaton, Marilyn A. Sharp, Tyson L. Grier, Keith G. Hauret, Bruce H. Jones, Mary L. Bouxsein, James P. McClung, Ronald W. Matheny, Susan P. Proctor

**Affiliations:** 1 Military Performance Division, United States Army Research Institute of Environmental Medicine, 10 General Greene Ave, BLDG 42, Natick, MA 01760 USA; 2 Department of Exercise Science, University of South Carolina, Columbia, SC USA; 3 Injury Prevention Division, United States Army Public Health Center, Aberdeen Proving Ground, MD USA; 4 Center for Advanced Orthopedic Studies, Beth Israel Deaconess Medical Center, Boston, MA USA; 5 Department of Orthopaedic Surgery, Harvard Medical School, Boston, MA USA; 6 Endocrine Unit, Massachusetts General Hospital, Boston, MA USA; 7 Military Nutrition Division, United States Army Research Institute of Environmental Medicine, Natick, MA USA

**Keywords:** Musculoskeletal injury, Military, Basic combat training, Stress fracture, Military readiness, ARMI study

## Abstract

**Background:**

Musculoskeletal injuries (MSKIs) are common in military trainees and present a considerable threat to occupational fitness, deployability, and overall military readiness. Despite the negative effects of MSKIs on military readiness, comprehensive evaluations of the key known and possible risk factors for MSKIs are lacking. The U.S. Army Research Institute of Environmental Medicine (ARIEM) is initiating a large-scale research effort, the ARIEM Reduction in Musculoskeletal Injury (ARMI) Study, to better understand the interrelationships among a wide range of potential MSKI risk factors in U.S. Army trainees in order to identify those risk factors that most contribute to MSKI and may be best targeted for effective mitigation strategies.

**Methods:**

This prospective study aims to enroll approximately 4000 (2000 male and 2000 female) U.S. Army trainees undergoing Basic Combat Training (BCT). Comprehensive in-person assessments will be completed at both the beginning and end of BCT. Participants will be asked to complete surveys of personal background information, medical history, physical activity, sleep behaviors, and personality traits. Physical measurements will be performed to assess anthropometrics, tibial microarchitecture and whole body bone mineral density, muscle cross-sectional area, body composition, and muscle function. Blood sampling will be also be conducted to assess musculoskeletal, genetic, and nutritional biomarkers of risk. In addition, participants will complete weekly surveys during BCT that examine MSKI events, lost training time, and discrete risk factors for injury. Participants’ medical records will be tracked for the 2 years following graduation from training to identify MSKI events and related information. Research hypotheses focus on the development of a multivariate prediction model for MSKI.

**Discussion:**

Results from this study are expected to inform current understanding of known and potential risk factors for MSKIs that can be incorporated into solutions that optimize Soldier health and enhance military readiness.

## Background

Musculoskeletal injuries (MSKIs) are common in military trainees and present a considerable threat to occupational fitness, deployability, and overall military readiness. In 2016, approximately 83% of all injuries experienced by U.S. Army Active Component Soldiers were musculoskeletal. These types of injuries, and related conditions, are the leading cause of outpatient medical encounters, lost duty days, and medical disability/discharge in the Active Component of the U.S. Army [1]. In the U.S. Army, MSKIs are particularly common during Basic Combat Training (BCT)—a time of heightened physical training at the beginning of a Soldier’s military career—with reports of MSKI incidence as high as 42% in men and 62% in women [2, 3]. Trainees who sustain an injury during initial military training are three times more likely to be discharged from the military compared to their non-injured counterparts [4]. Interventions or other risk mitigation strategies to prevent medical discharge could have an estimated cost avoidance of over $50,000 per trainee [5].

**Fig. 1:**
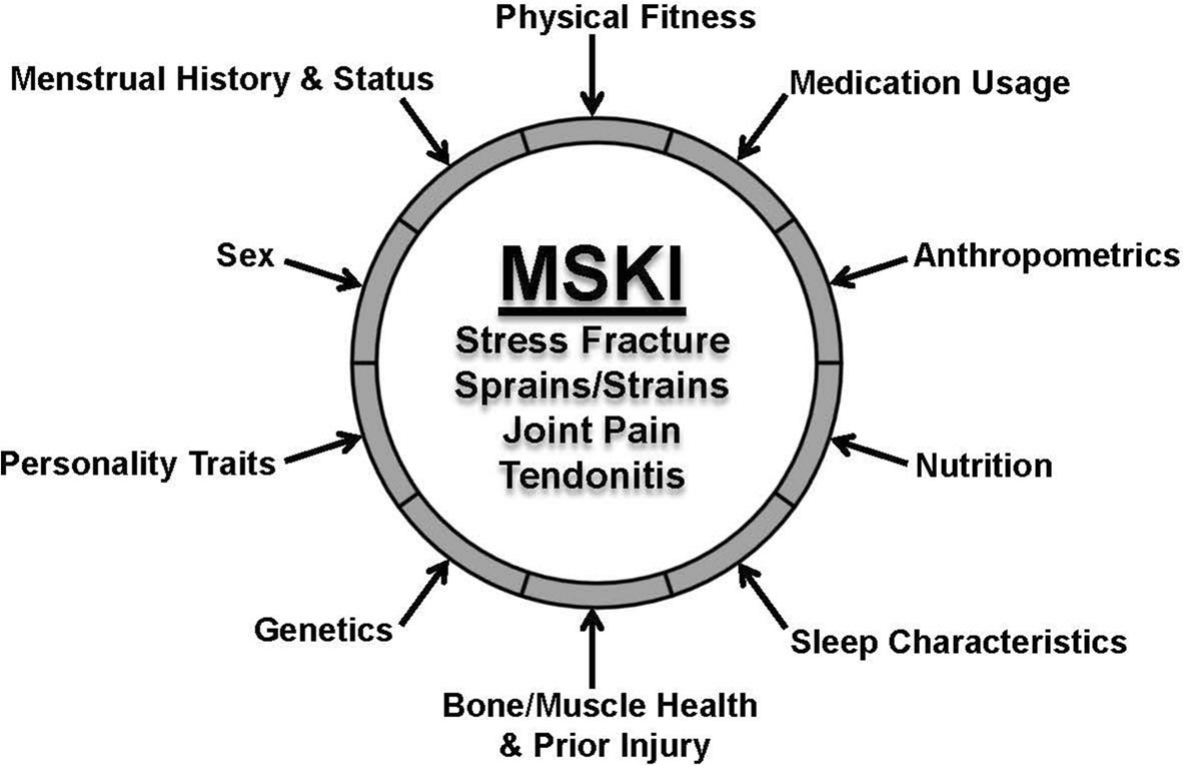
By concurrently assessing multiple established and emerging risk factors for MSKIs in trainees undergoing Basic Combat Training (list is a sampling of factors to be investigated), the ARMI Study aims to identify which factors are most predictive of injury risk

Common MSKIs during military training include stress fractures, strains, sprains, tendinitis, and overuse knee injuries [2]. The etiology of distinct MSKIs may vary, but these injuries share several established risk factors such as age, sex, prior injury risk, physical activity history, physical fitness, and smoking history [2, 3, 6–18]. Evidence for other potential predictive risk factors such as use of pain management and hormonal contraceptive medications, bone microarchitecture and muscle cross-sectional area, sleep characteristics, personality traits, dietary intake, genetics, and performance on physical fitness tests is much more limited (Fig. 1). Recently, in an effort to mitigate the effects of MSKI on service members, the U.S. Army has initiated several injury prevention initiatives, including a pre-enlistment physical screening test [19] and a calcium and vitamin D-fortified snack bar [20]. At present, the relative contributions of established and potential/emerging risk factors, as well as these newly-introduced injury prevention initiatives, remain unclear. Moreover, much of the research examining these risk factors has focused on individual factors rather than evaluating the effects of multiple factors concurrently. Finally, many of these factors remain to be investigated over a span of time that includes the beginning of training, regularly throughout training, at training completion, and for an extended follow-up period. Accordingly, The U.S. Army Research Institute of Environmental Medicine (ARIEM) is initiating a large-scale research effort, the ARIEM Reduction in Musculoskeletal Injury (ARMI) Study, to address these research gaps.

Beyond investigation of factors that influence MSKI, the ARMI Study also aims to identify the impacts of these factors on musculoskeletal physiology during BCT. Investigation of bone and muscle adaptation to BCT is of interest in this study due to the physiological responsiveness of these connective tissues to military training as reported in prior studies [20–23], which may allow for identification of physiological mechanisms underlying MSKI.

The overarching goals of this study, therefore, are to build a predictive model of risk factors for MSKI, to identify trainees at greatest risk for injury, and to identify potential mechanisms of injury and modifiable risk factors that can be targeted for intervention. Such results will provide the foundations for evidence-based recommendations to military leaders and medical personnel to reduce MSKIs in military trainees and therefore improve post-training military readiness.

## Methods/design

### Project aims

This research effort has three primary aims: 1) identify novel risk factors for MSKI; 2) evaluate the effectiveness of ongoing MSKI prevention initiatives within the U.S. Army Basic Combat Training environment; and 3) develop a predictive model to identify trainees at greatest risk of MSKI.

### Design

This is a prospective, longitudinal cohort study of U.S. Army trainees undergoing BCT. The study has received approval from the U.S. Army Medical Research and Materiel Command Institutional Review Board (Protocol Number M-10678) and the U.S. Army Training and Doctrine Command’s Center for Initial Military Training.

### Setting

This research involves on-site data collection from the four U.S. Army Basic Combat Training bases: Ft. Jackson, SC., Ft. Benning, GA, Ft. Sill, OK, and Ft. Leonard Wood, MO.

### Participants

Participants will be recruited from incoming classes of male and female trainees. Approximately 4000 trainees (2000 women and 2000 men) will be enrolled. Participants are able to enroll in the study if they meet the following criteria:

Inclusion Criteria: Any U.S. Army trainee who is at least 17 years old.

Exclusion Criteria:

1. Individuals who are older than 42 years of age.
2. Individuals who are restricted from performing physical activity.
3. Individuals who have any self-identified chronic or recent injuries/illnesses that limit physical activity.
4. Individuals who self-identify as having a history of bone-modifying disorders (e.g. osteogenesis imperfecta, osteopetrosis, or rickets).
5. Individuals who self-identify as having an endocrine disorder (e.g. diabetes, hypoparathyroidism, or hyperparathyroidism).
6. Individuals who report they are currently taking or have taken oral glucocorticoid drugs (e.g. prednisone or hydrocortisone) in the two years prior to the study.
7. Individuals who are pregnant or breastfeeding.
8. Individuals who have metal in their body in regions that may interfere with the quality of the bone scans (typically from prior injury in the form of rods, plates, pins, etc.).

### Study overview

**Fig. 2:**
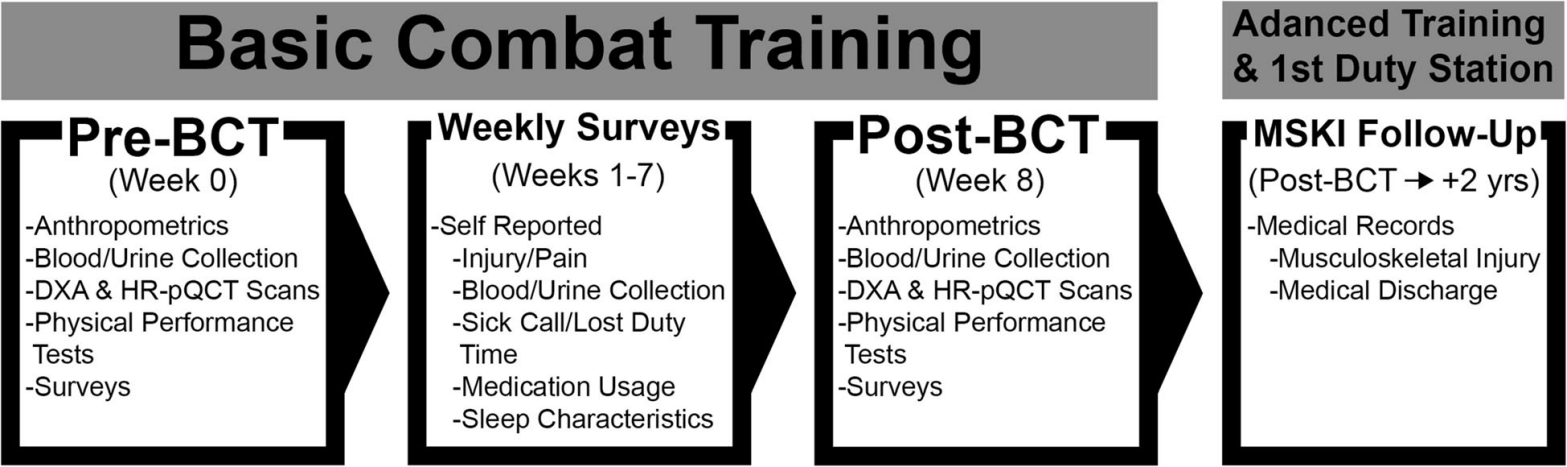
Study Overview. *BCT* Basic Combat Training, *DXA* Dual-Energy X-ray Absorptiometry, *HR-pQCT* High Resolution Peripheral Quantitative Computed Tomography

In-person data collection activities will commence during the first week of BCT and continue once each week during the eight weeks of BCT, concluding in the final week of BCT, prior to graduation (Fig. 2). Data from medical and training records will also be collected during BCT and for two years after graduation.

### Research procedures

#### Anthropometrics and body composition testing

Participants will have height and weight measured while in standardized physical training (PT) uniforms (athletic shorts and t-shirt) and without shoes. Height, weight, and body circumference measurements will be taken at the beginning and end of BCT. Circumference measurements will be taken at the neck and waist for males, and at the neck, waist, and hips for females to estimate body fat percent, per U.S. Army regulation 600.9 [24].

Body composition will be assessed by dual-energy X-ray absorptiometry (DXA) using a Lunar Prodigy system (GE Healthcare, Madison, WI) at the beginning and end of BCT to assess the effects of training on body fat and lean mass. A quality control phantom will be scanned daily to ensure the equipment is calibrated before each data collection session. Total body estimates of percent fat, bone mineral density, and body content of bone, fat, and non-bone lean tissue will be determined using manufacturer described procedures and supplied algorithms (Encore, version 11.40, Lunar Corp., Madison, WI).

#### Tibial bone microarchitecture and calf muscle morphology

Bone density and microarchitecture will be assessed at the distal and/or mid-shaft tibia in participants using a high-resolution peripheral quantitative computed tomography (HR-pQCT) system (XtremeCT II, Scanco Medical AG®, Brütisellen, Switzerland) at the beginning and end of BCT to assess baseline bone and calf-muscle characteristics and changes in these characteristics over the course of the training period. HR-pQCT technology acquires computed tomography slices with an isotropic voxel size of 61 μm^3^, allowing for assessment of bone density and microarchitecture in vivo*.* The region of interest to be scanned will be identified from a 2D scout view by placing a reference line at the distal endplate of the tibia. HR-pQCT measurements will be performed on the non-dominant leg, unless there is a history of fracture in the tibia, in which case the non-fractured leg will be measured [22]. The outcome variables computed by automated analysis include muscle cross-sectional area (mm^2^); intramuscular fat (% of muscle area); bone volume/total volume; volumetric bone mineral density (mg hydroxyapatite/cm^3^) for the total, trabecular, and cortical regions; trabecular number (mm^− 1^), thickness (μm), and separation (μm); and cortical thickness (μm) and porosity (%) [25].

#### Physical performance testing

A series of physical performance tests will be administered during each iteration to assess muscle function. Before and after BCT, maximum vertical jump height will be used to determine lower extremity muscle power [26]. Three additional tests will be used to quantify baseline muscle function at entry to BCT. To determine lower extremity endurance, trainees will perform single-leg wall squats for as long as possible, up to 60 s [27]. Lower extremity symmetry and functional stability will be assessed using the star excursion test [28], which requires trainees to balance on one leg while reaching as far as possible with the other leg in 8 directions. Finally, ankle range of motion and calf flexibility will be measured by having participants complete a weight-bearing lunge test for distance [29].

#### Surveys

A series of surveys will be administered to participants throughout BCT. The purpose of these surveys is to obtain information regarding individual characteristics and behaviors.

### Background and exit surveys

The background survey will be administered during data collection in the first week of BCT. This survey consists of questions pertaining to the participants’ demographics, physical activity history, injury history, medication and supplement use (e.g., NSAIDs, hormonal contraceptives, multivitamins, etc.) and menstrual cycle history. The exit survey will be administered during the final week of BCT and will include questions similar to the background survey but in reference to the participants’ BCT experience.

### Sleep surveys

Sleep surveys, modified from the Pittsburgh Sleep Quality Index (PSQI) [30], the Epworth Sleepiness Scale [31], and the Morningness-Eveningness questionnaires [32], will be administered at the beginning and end of BCT and will consist of questions that assess the quantity and quality of sleep of the participants, as well as identify their sleep patterns [30].

### Personality characteristics, nutrition, and pain surveys

Additional surveys will be administered both before and after BCT to assess personality traits (e.g. hardiness, grit), average consumption of calcium and vitamin D rich foods, and pain experience and management. These surveys include or are adaptations of the Angela Duckworth Grit Scale [33], the Dispositional Resilience Scale-II [34], the Pain-Catastrophizing Scale [35], the Brief Pain Inventory-SF [36], and a calcium and vitamin D specific survey from NutritionQuest [37].

### Weekly survey

The weekly surveys will assess receipt of and/or adherence to supplements currently offered in BCT (e.g. multivitamin with iron, Performance Readiness Bar) and prescription medications (e.g. NSAIDs and hormonal contraceptives), sleep quantity and quality, new or persistent sickness, injuries and pain, and missed training activities associated with sickness or injury during each specific week of BCT.

#### Biological samples

Fasted blood samples (approximately 35 mL per draw, approximately 70 mL total) will be collected from venous blood at the beginning and end of BCT for assessment of biochemical markers of bone formation and resorption, biochemical markers of metabolism, and genetic biomarkers. Morning void urine sample will also be collected to screen for pregnancy in female participants.

#### Army records data

Graduation, attrition, physical fitness (Occupational Physical Assessment Test (OPAT) and Army Physical Fitness Test (APFT) scores), and medical encounter data will be collected from the training unit and through collection of Army records by the U.S. Army Public Health Center (APHC).

### Graduation/attrition

Graduation dates or attrition dates and reason the trainee left the unit will be recorded. APHC will also obtain information on Soldier attrition (date and reason for attrition) both during and after BCT using the Army Training Resources and Requirements System (ATRRS), the Army Management Information System of Record for managing student input to training.

### Army fitness tests

The Occupational Physical Assessment Test (OPAT) is given during the accession process to assess physical competency for an individual’s assigned career field and consists of a standing broad jump, seated medicine ball put, dead lift, and timed shuttle run [19]. Raw scores from the participants' final OPAT will be obtained from the unit, or requested directly from the U.S. Army Training and Doctrine Command (TRADOC).

The Army Physical Fitness Test (APFT) is the Army’s standardized test for overall fitness. Baseline fitness data may include 1-min push-ups and sit up scores, as well as a 1-mile run time. End of training test data will consist of 2-min push-up and sit-up scores as well as 2-mile run times. Raw APFT scores will be obtained at the beginning and ending of BCT from the training units.

### Medical encounter data

Medical encounter data are entered into a trainee’s official medical record for each medical visit to an Army healthcare provider. Such data include reasons for visit, diagnoses received, tests administered, and prescriptions written and filled. These data will be obtained from the Defense Medical Surveillance System (DMSS) which is maintained by the Armed Forces Health Surveillance Branch, Defense Health Agency (DHA) and/or from the Medical Data Repository with oversight from the DHA. Additionally, pharmacy prescription data will be obtained from the DMSS or the DHA Pharmacy Analytics Support Sections Integrated Utilization Branch Pharmacy Operations Divisions. All requests for medical encounter and pharmacy data will be coordinated by the USAPHC through the DHA.

#### Power calculation

The goal of this prospective observational study is to recruit up to 4000 (2000 males and 2000 females) participants to have sufficient power (≥80%) to detect a statistically significant effect (alpha < 0.05) based on the study design, analysis plan, and an anticipated drop out and loss- to-follow-up of 25% over the 8 weeks. To calculate power and sample size, an approach using multiple study simulations was employed where the percentage of simulated studies that produce a *p*-value < 0.05 for the relationship of interest would be an estimate of the study power [38]. Within the simulated population, the exposure and outcome distributions for this study were defined and the expected effect size related to the relationship between the predictors and outcomes was accounted for. Monte Carlo methods were used to randomly select individuals from this hypothetical population to create 1000 pseudo study populations. These pseudo populations were used to evaluate our proposed models and to produce *p*-values indicating the statistical significance between the proposed relationship of the predictors and outcome of interest [39]. Each study simulation was run, increasing the sample size of the individuals selected for the study population until the minimum threshold power of 80% was reached for all primary outcomes, including MSKIs and indices of musculoskeletal adaptation to training.

#### Statistical analyses

Prediction models will be created that take into consideration both modifiable and non-modifiable risk factors as potential predictors of MSKI and bone and muscle adaptation to training. Linear and non-linear relationships between these outcomes and the individual covariates will be explored using both linear models and generalized additive models with naturalized splines. Multiple imputation will be used to handle missing data. Model selection and internal validation will be made using a Monte Carlo cross validation in order to minimize the potential for overfitting the model and to increase the accuracy of prediction so that final results may be applicable to the entire Army.

## Discussion

The approach of examining a comprehensive inventory of modifiable and non-modifiable risk factors and tracking injury and related outcomes in a large cohort of trainees during BCT will allow for identification of those at greatest risk for MSKI. While non-modifiable factors such as sex and race/ethnicity may help identify trainees at greatest risk of injury, modifiable factors such as physical fitness, medication usage, and dietary habits can be targeted for intervention. By also investigating musculoskeletal adaptation to training, mechanisms of MSKI can be explored, providing novel evidence for the physiological underpinnings of injuries, and potentially, targets for future interventions. Results from this study may be applicable to civilian populations at risk for MSKI, such as those participating in repetitive physical activities like endurance running. However, the ultimate goal of the study is to provide data from military trainees that can be incorporated into solutions for use by military leaders and medical personnel to effectively reduce MSKI incidence, decrease medical costs, and enhance military readiness.

## Abbreviations

APFT: Army Physical Fitness Test
APHC: Army Public Health Center
ATRRS: Army Training Requirements and Resource System
BCT: Basic Combat Training
DXA: Dual-Energy X-ray Absorptiometry
HR-pQCT: High Resolution Peripheral Quantitative Computed Tomography
MSKI: Musculoskeletal Injury
OPAT: Occupational Physical Assessment Test
TRADOC: Training and Doctrine Command
